# Critical care readiness in conflict and disaster-affected Somalia: mass-casualty response, emergency referral, and continuity of life-saving care

**DOI:** 10.1186/s13054-026-06258-5

**Published:** 2026-08-18

**Authors:** Ahmed Omar Siyad, Abdukadir Mohamed Hassan

**Affiliations:** Department of Critical Care Medicine, Dr. Sumait Hospital, SIMAD University, Mogadishu, Somalia

## Abstract

Critical care readiness during war and disasters is often judged by intensive care unit capacity, yet preventable mortality in fragile health systems may occur before, during, or after ICU-level care. In Somalia, recurrent mass-casualty events, emergency referral constraints, limited oxygen and blood readiness, constrained operative access, and weak post-resuscitation monitoring expose a critical gap between first contact and sustained life-saving care. This Comment addresses that gap by reframing critical care readiness in conflict and disaster-affected Somalia as a pathway problem rather than an ICU-capacity problem alone. Its novelty lies in integrating domains often treated separately—mass-casualty response, emergency referral, essential emergency and critical care inputs, ethical triage, and non-punitive system learning—into one practical readiness agenda for fragile health systems. The article argues that training and hospital preparedness are necessary but insufficient unless supported by referral communication, command structures, oxygen systems, blood readiness, ward monitoring, and clear escalation criteria. Strengthening this emergency-to-critical-care pathway can improve continuity of life-saving care, support fair resource allocation, and enhance accountability in health systems exposed to conflict and disasters.

## From ICU capacity to emergency-to-critical-care pathway readiness

Critical care in war and disaster areas is often framed in terms of intensive care unit (ICU) capacity; however, access to critical care medicine extends beyond ICU admission and depends on timely integrated emergency, critical and operative care across the pathway of acute illness and injury [1, 2]. In fragile health systems, the more decisive question is whether a patient with time-sensitive injury or acute illness can move safely through the full chain of life-saving care. The World Health Assembly emphasized emergency care as an integrated platform for timely care across acute illness and injury, including disasters, fragile settings, mass-casualty events and conflict-affected areas [1]. Its later mandate for integrated emergency, critical and operative care reinforced the need to protect life-saving services before, during and after health emergencies [2]. Somalia illustrates why this pathway view matters: a critically injured patient may die because timely emergency and critical care is inaccessible, triage is delayed, referral is unsafe, oxygen or blood is unavailable, operative access is constrained, or monitoring fails after initial resuscitation [3, 4].

## Somalia’s readiness gap

Recent national evidence shows that emergency and critical care (ECC) readiness in Somalia remains severely limited. A nationwide assessment of 131 hospitals reported a median ECC readiness score of 0.31, and more than 80% of assessed hospitals were not considered ready to provide ECC services using the study threshold [3]. Earlier World Health Organization (WHO) assessment work described gaps in designated emergency areas, round-the-clock emergency services, triage capacity, referral infrastructure, oxygen availability and blood services [4].

Somalia’s trauma evidence strengthens the case for an integrated readiness agenda. A retrospective cohort of 2426 patients injured by explosions and firearm attacks found that explosion injuries were associated with more severe and critical injuries than firearm wounds, that approximately one-quarter of patients required ICU care, and that inpatient mortality was 11.6% [5]. A study of mass-casualty incidents in Mogadishu from 2013 to 2018 reported that mechanism of injury and bleeding were significantly associated with mortality [6]. After the 29 October 2022 double bombing in Mogadishu, WHO reported more than 117 deaths, over 344 injuries and approximately 70% of trauma deaths before hospital arrival [7]. These findings indicate that preventable deaths may occur at the interfaces between scene response, referral, resuscitation, surgery and continuing critical care.

## The fragmentation problem

The central gap addressed here is fragmentation alongside scarcity. Emergency care systems are intended to connect prehospital recognition, transport, emergency-unit care, early operative care and critical care [8]. Available evidence and operational experience suggest that mass-casualty response planning, ambulance referral, hospital triage, operating-theatre access and ICU support in Somalia remain insufficiently integrated [3, 4, 9]. A recent evaluation of the WHO Mass Casualty Management course in Somali trauma hospitals reported improved knowledge and self-reported confidence after training, supporting structured preparedness education, role clarity and hospital-specific planning [9]. Training remains necessary, but its effect is likely to be limited unless referral communication, command structures, oxygen access, blood readiness and staffing are strengthened. The priority is to convert episodic preparedness into a coordinated emergency-to-critical-care pathway. Table 1 summarizes four domains where this shift can be operationalized.

**Table 1:** Readiness priorities for conflict and disaster-affected Somalia

Readiness domain	Current risk in conflict and disaster settings	Priority action for Somalia	Expected contribution to SDG 3 and SDG 16
Mass-casualty response	Hospitals may be overwhelmed when casualties arrive without coordinated triage, roles, tracking or surge plans.	Use mass-casualty plans, incident-command roles, triage zones, documentation tools and drills.	Improves timely care and strengthens preparedness and accountability.
Emergency referral	Patients may arrive late, deteriorate in transit or reach facilities without needed capacity.	Create referral protocols, pre-arrival communication, destination rules and stabilization standards.	Reduces avoidable mortality and supports equitable access.
Continuity of life-saving care	Initial resuscitation may not be followed by oxygen, monitoring, transfusion, surgery or critical care nursing.	Prioritize ECC inputs, oxygen systems, blood readiness, ward monitoring and ICU step-up criteria.	Protects critically ill patients across the pathway.
Ethics and governance	Crisis decisions may be inconsistent when scarce resources are under pressure.	Develop transparent triage ethics, fair allocation, mortality review and non-punitive learning.	Strengthens trust, fairness and institutional legitimacy.

## Operational priorities

Mass-casualty response should be a routine hospital capability rather than an improvised reaction. This requires visible command roles, triage areas, patient-flow mapping, communication with ambulance services and security actors, and documentation systems that remain usable when electricity, internet and staffing are disrupted. Global emergency care guidance identifies triage, checklists, governance, training and coordination with disaster-response actors as essential emergency-system functions [1]. In Somalia, these processes must be low-cost, repeatedly drilled and adapted to hospitals receiving explosions, firearm injuries, road-traffic trauma, floods and displacement-related emergencies.

Emergency referral should be treated as a clinical intervention. Referral is not merely transport; it is a risk-bearing process requiring stabilization, destination selection, pre-arrival notification and structured handover. Disease Control Priorities describes emergency care as a system spanning prehospital care and transport through emergency-unit care to early operative and critical care [8]. Referral protocols should clarify whether the patient is stable enough to move, which facility has the required capability, whether the receiving team is ready, and what treatment must continue during transfer.

Continuity of life-saving care should be defined around essential functions rather than advanced technology alone. Early critical care in low-resource settings should prioritize recognition of deterioration, airway and breathing support, oxygen therapy, circulation support, infection treatment, pain control, monitoring, nursing observation and escalation to operative or intensive care when needed [10, 11]. Oxygen is central because hypoxaemia cuts across trauma, sepsis, pneumonia, perioperative care and obstetric emergencies. Global work argues that oxygen investments should be embedded within essential emergency and critical care rather than treated as equipment procurement alone [12]. Somalia-specific research on solar-powered oxygen delivery suggests that adapted oxygen systems can be feasible when electricity access is unreliable [13].

## Ethics and system learning

Ethics must be placed at the center of readiness. In mass-casualty events, scarcity can force decisions about oxygen, blood, operative timing, ICU admission and transport priority. Ethical triage should be supported by transparent protocols, multidisciplinary review and institutional accountability, not left to individual clinicians under pressure. The World Health Assembly urges safe, high-quality, needs-based emergency care without discrimination or payment barriers before care is provided [1]. In Somalia, these principles are part of clinical ethics, public trust and Sustainable Development Goal (SDG) 16. Non-punitive mortality review after mass-casualty incidents should identify where the pathway failed, whether in scene response, referral, triage, oxygen access, operative delay, ICU admission or ward monitoring.

## Conclusion

Critical care readiness in conflict- and disaster-affected Somalia should be reframed from a narrow focus on ICU capacity to an integrated pathway for mass-casualty response, emergency referral and continuity of life-saving care. Accordingly, hospitals and health authorities should establish written mass-casualty and referral protocols, conduct regular drills, require pre-arrival notification and structured handover, maintain reliable oxygen systems and blood availability, define ward-to-ICU escalation criteria, and conduct multidisciplinary, non-punitive mortality reviews after major incidents. The central priority is to ensure that critically ill and injured patients are not lost between first contact, referral, stabilization, and sustained life-saving care.

## Use of large language models

During manuscript preparation, ChatGPT was used for language polishing and formatting support. The authors verified all content, citations, and references, approved the final manuscript, and remain fully responsible for the work.

## Data Availability

No datasets were generated or analysed during the current study.

## Abbreviations

ECC: Emergency and critical care
ICU: Intensive care unit
SDG: Sustainable Development Goal
WHO: World Health Organization
